# Principles and properties of ion flow in P2X receptors

**DOI:** 10.3389/fncel.2014.00006

**Published:** 2014-02-05

**Authors:** Damien S. K. Samways, Zhiyuan Li, Terrance M. Egan

**Affiliations:** ^1^Department of Biology, Clarkson UniversityPotsdam, NY, USA; ^2^Guangzhou Institute of Biomedicine and Health, University of Chinese Academy of SciencesGuangzhou, China; ^3^Department of Pharmacological and Physiological Science, The Center for Excellence in Neuroscience, Saint Louis University School of MedicineSt. Louis, MO, USA

**Keywords:** P2X, ATP, permeability, selectivity, gating, mutagenesis, SCAM

## Abstract

P2X receptors are a family of trimeric ion channels that are gated by extracellular adenosine 5′-triphosphate (ATP). These receptors have long been a subject of intense research interest by virtue of their vital role in mediating the rapid and direct effects of extracellular ATP on membrane potential and cytosolic Ca^2+^ concentration, which in turn underpin the ability of ATP to regulate a diverse range of clinically significant physiological functions, including those associated with the cardiovascular, sensory, and immune systems. An important aspect of an ion channel's function is, of course, the means by which it transports ions across the biological membrane. A concerted effort by investigators over the last two decades has culminated in significant advances in our understanding of how P2X receptors conduct the inward flux of Na^+^ and Ca^2+^ in response to binding by ATP. However, this work has relied heavily on results from current recordings of P2X receptors altered by site-directed mutagenesis. In the absence of a 3-dimensional channel structure, this prior work provided only a vague and indirect appreciation of the relationship between structure, ion selectivity and flux. The recent publication of the crystal structures for both the closed and open channel conformations of the zebrafish P2X4 receptor has thus proved a significant boon, and has provided an important opportunity to overview the amassed functional data in the context of a working 3-dimensional model of a P2X receptor. In this paper, we will attempt to reconcile the existing functional data regarding ion permeation through P2X receptors with the available crystal structure data, highlighting areas of concordance and discordance as appropriate.

## Introduction

The P2X receptors are a family of seven (P2X1R–P2X7R) cation permeable ligand-gated ion channels (LGICs) that open in response to binding by the extracellular ligand, adenosine 5′-triphosphate (ATP). In contrast to tetrameric ionotropic glutamate receptors and pentameric Cys-loop receptors, the P2XRs are assembled from three peptide subunits (Nicke et al., 1998; Stoop et al., 1999; Jiang et al., 2003; Barrera et al., 2005). Each subunit is comprised of intracellular amino and carboxyl termini linked via two transmembrane-spanning helices (TM1 and TM2) to a large extracellular ligand-binding domain (Newbolt et al., 1998; Torres et al., 1998a,b). Binding of ATP to a site in the extracelluar domain elicits a global conformational change that ultimately leads to the opening of a pore through which cations freely move into and out of the cell (Baconguis and Gouaux, 2012; Hattori and Gouaux, 2012). With the exception of P2X6R, all the subunits assemble into functional homomeric ion channels. In addition, several heteromeric assemblies have been identified and characterized, including the P2X2/3R functionally expressed in pain-processing neurons (Lewis et al., 1995; Le et al., 1998; Torres et al., 1998c, 1999; King et al., 2000; Aschrafi et al., 2004; Compan et al., 2012). P2XRs exhibit little discrimination between Na^+^ and K^+^ but, at resting membrane potentials, currents are chiefly carried by movement of Na^+^ down its electrochemical gradient and into the cell. The result is membrane depolarization. All P2XRs also conduct Ca^2+^, with the permeability of Ca^2+^ relative to Na^+^ (P_Ca_/P_Na_) varying depending on the subunit make-up of the functional channel (Egan and Khakh, 2004). Thus, the two initial consequences of P2XR activation to cellular signaling are a Na^+^-mediated depolarization of the plasma membrane, and an increase in the concentration of free cytosolic Ca^2+^ ([Ca^2+^]_*i*_). These two results subsequently influence action potential propagation and affect a myriad of Ca^2+^-sensitive processes, including secretion (Khakh and Henderson, 2000; Norenberg et al., 2001), muscle contraction (Lamont and Wier, 2002; Brain et al., 2003), and cell survival (for review see Di Virgilio, 2012; Volonte et al., 2012).

**Figure 1:**
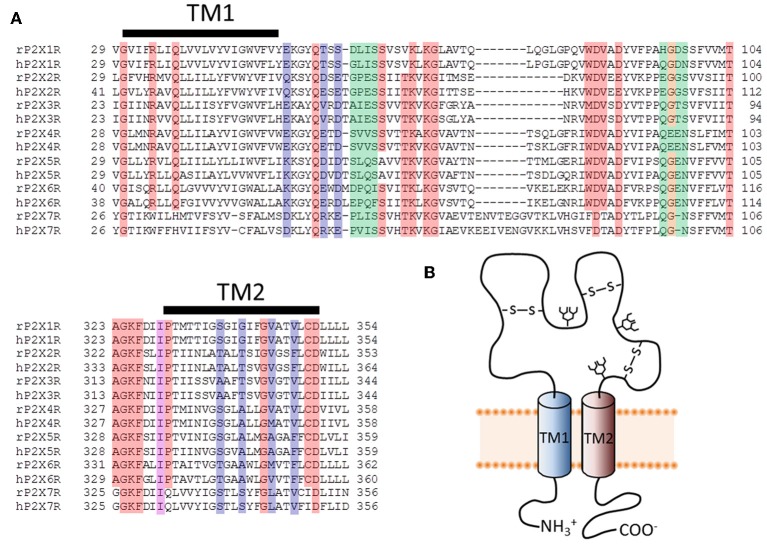
**Primary structure of putative pore-forming domains of P2XR subunits. (A)** Sequence alignments for select stretches of P2XR subunit polypeptides that include the two transmembrane domains (TM1 and TM2). Both human and rat forms of the P2XRs are shown. Red, orange, and purple boxes indicate highly conserved amino acids that appear in at least six of the seven P2XRs. Blue and purple boxes indicate amino acids likely to form the principal ion permeation pathway of the lateral portals and transmembrane spanning pore. Green and orange boxes indicate residues lining the central and upper vestibules, but which do not appear to form the principal ion permeation pathway. **(B)** Schematic representation of a single P2XR subunit, showing the intracellular amino and carboxyl termini linked by two TM domains and a large ligand-binding extracellular domain.

Over the last two decades, an exhaustive effort to relate P2XR structure to the function of these channels has provided a good understanding of how ATP transduces current across the plasma membrane. Multiple laboratories have successfully utilized molecular biological techniques and patch clamp electrophysiology to identify amino acids necessary for ligand binding, signal transduction and ion permeation. Nevertheless, the successful crystallization of a zebrafish P2X4.1R, in both the closed (Kawate et al., 2009; PDB ID 4DW0) and ATP-bound open (Hattori and Gouaux, 2012; PDB ID 4DW1) conformations was a dramatic advance for the field. On the one hand, these structures serve as the basis of a slew of new testable hypotheses about the relationship between P2XR structure and function. On the other, they provide an all-important 3-dimensional template upon which to review and interpret previously obtained functional data. Many of the most important advances are described in numerous reviews (Egan et al., 2006; Khakh and North, 2006; Roberts et al., 2006; Burnstock and Kennedy, 2011; Coddou et al., 2011; Kaczmarek-Hajek et al., 2012; North and Jarvis, 2013). This review focuses entirely on the following two questions: (1) How do ions enter the pore of a P2XR and subsequently transition from one side of the plasma membrane to the other? (2) How do P2XRs discriminate between ions, selecting and permeating some to a greater degree than others? With these questions in mind, the principle objective of this review is to view the data obtained from functional studies over the last two decades within the context of the now available 3-dimensional crystal structures, particularly that of the open channel state, in order to gauge the degree of concordance and potentially identify areas of inconsistency. As a visual aid, a number of figures are included which serve to simply map the results of various systematic functional studies onto the relevant P2XR structure. We have also included a sequence alignment showing examples of human and rat P2XRs for reference (Figure 1). Most of the studies investigating ion permeation and selection in this family of ion channels were conducted on the P2X2R and P2X4R. Homology models for these receptors were generated based on the available crystallographic data obtained for the truncated zebrafish P2X4.1R.

## Extracellular access to the transmembrane channel pore

**Figure 2:**
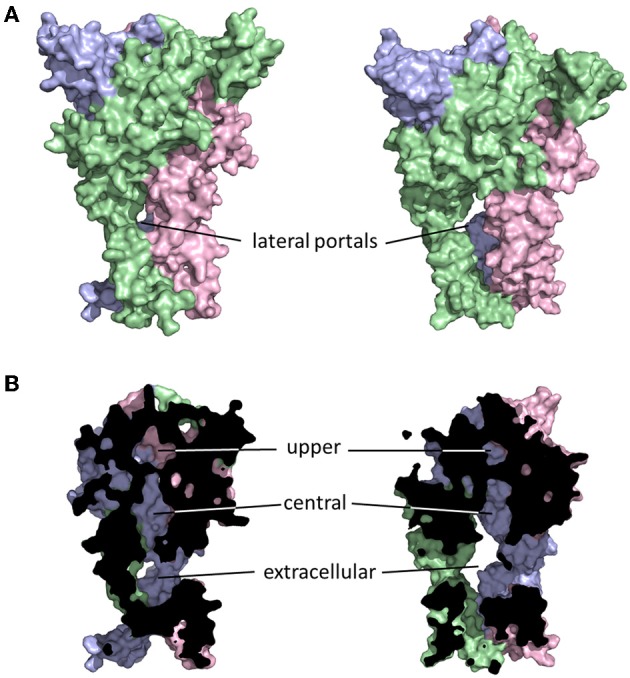
**Ion access to the transmembrane pore of a P2XR**. Shown are closed (left) and open (right) P2X2R homology models built based on the resolved structures for zebrafish P2X4R (Kawate et al., 2009; Hattori and Gouaux, 2012). Subunits are presented in different colors. The lateral portals are labeled in the complete structure panels presented in **(A)**. The three internal cavities within the extracellular domain of the P2XR are shown in the cutaway models of **(B)**.

The initial publication of the closed channel structure for the zebrafish P2X4R revealed the presence of three lateral portals, or fenestrations, situated in the extracellular domain proximal to the outer leaflet of the lipid bilayer (Kawate et al., 2009). The diameter of these lateral portals, equal to ~12Å, is sufficient to allow for the passage of water and fully hydrated ions, and provoked the compelling hypothesis that they might serve as the primary access points for extracellular ions to approach the mouth of the transmembrane pore itself. The closed structure also indicated the presence of three cavities within the extracellular domain of the receptor, which Kawate et al. (2009) designated the upper, central and extracellular vestibules (Figure 2). These form a broken chain along the central axis of the receptor, and present an alternative ion conduction hypothesis in which the central pathway widens during gating to form a single pore running the entire length of the protein. Further, Kawate et al. saw a gadolinium ion (Gd^3+^) coordinated in the central vestibule of zebrafish P2X4.1R, and used patch clamp electrophysiology to show that Gd^3+^ inhibited ATP-gated current. However, it did so in a manner that was surmountable by increasing the concentration of ATP, suggesting that the inhibition of current was not caused by Gd^3+^ occluding an ionic conduction pathway. Indeed, the fact that current still flowed through the P2X4.1R even when the central vestibule was inhibited by a Gd^3+^ ion suggested that ion entry occurs below this point in the structure. Furthermore, the closed structure shows at least two barriers to ion movement along the central pathway that would have to open in order to allow permeation. The presence of extracellular gates that impede ion flow was not predicted from the results of numerous experiments that used the Scanning Cysteine Accessibility Method (SCAM) to identify differences in solvent accessibility of open and closed channels (Egan et al., 1998; Jiang et al., 2001; Kracun et al., 2010; Li et al., 2010). Indeed, the use of the SCAM in P2X2R and P2X4R revealed that cysteine substitutions introduced into the upper vestibule were not rapidly accessible to modification by thiol-reactive methanethiosulfonate (MTS) compounds (Kawate et al., 2011; Samways et al., 2011). Kawate et al. (2011) produced a double-cysteine mutant P2X2R containing a disulfide bond across the central axis of the central vestibule, which did nothing to impair ion flow. To the contrary, currents through the channel were inhibited rather than potentiated by reduction of the S-S bond with dithiothreitol (DTT) suggesting that while movement in this region might be important for gating, it is highly unlikely that it contributes to the ion conductions pathway.

The lateral pore hypothesis was largely validated by the publication of the ATP-bound open channel structure of zfP2X4R (Hattori and Gouaux, 2012). As the rat P2X2R homology model in Figure 2 suggests, these lateral portals expand during gating, in agreement with predictions made on the basis of functional experiments (Kawate et al., 2011; Samways et al., 2011). In the open structure state, the upper vestibule seems to remain isolated from the bulk solution, but the central and extracellular vestibules appear to merge to form a single large inner cavity that extends into the opened transmembrane ion conducting pathway. The merging of the central and extracellular vestibules concords with functional data, with cysteine substitutions introduced at positions within the central vestibule in P2X1R, P2X2R, and P2X4R being accessible to water-soluble thiol-reactive agents in the open state. Specifically, currents through P2X1R-G60C, P2X2R-I317C, and P2X2R-H319C, and P2X4R-S62C and P2X4R-N97C, were all modified by the positively charged thiol-reactive MTS reagent, MTSET^+^ (Kawate et al., 2011; Samways et al., 2011). However, the currents through four of these mutants, P2X1R-G60C, P2X2R-H319C, P2X4R-E56C, and P2X4R-D58C, were potentiated rather than suppressed by MTSET^+^ modification.

The central vestibule has a negative electrostatic surface potential (Kawate et al., 2009, 2011). It also shows some charge discrimination, as seen in its ability to discriminate between cationic and anionic MTS compounds. Brief 3 s exposure to the anionic MTS reagent, MTSES^−^, had no effect on currents mediated by P2X4R-S62C and P2X4R-N97C, and more importantly did not prevent subsequent MTSET^+^ exposure from potentiating currents (Samways et al., 2011), indicating that only the positively charged molecule could access and modify these sites. On the other hand, much longer applications (~60 s) were sufficient for MTSES^−^ to modify cysteines introduced in the same region of P2X2R (Jiang et al., 2010). Nevertheless, MTSES^−^ can readily and rapidly modify cysteines introduced intracellular to this region within the lateral portals and as far down into the transmembrane pore as positions Ser^341^ in P2X4R (Samways et al., 2011) and Thr^336^ in P2X2R (Rassendren et al., 1997), adding weight to the idea that thiol-reactive agents enter the extracellular vestibule through the lateral portals.

The possibility that P2XRs might inhabit conformational states in which ions can intrude into the central pathway cannot be completely ruled out. SCAM studies of P2X1R showed that longer (5–60 min) incubations with thiol-reactive reagents were sufficient to cause modification of upper vestibule positions (Allsopp et al., 2011; Roberts et al., 2012a), indicating that these areas are not completely sealed from the surrounding aqueous environment. Based on free energy calculations and the closed zebrafish P2X4.1R crystal structure, Kawate et al. (2011) estimated that a modest widening of the central axis pore during gating could feasibly provide a sufficient permeation pathway favorable for Na^+^ conduction, but found that this was not so for the P2X2R homology model due to differences in primary amino acid sequence within this region of the receptor. Along similar lines, Roberts et al. (2012a,b), showed that the cysteines introduced into the upper vestibule of P2X1R were more easily accessed and modified by N-Biotinoylaminoethyl-MTSEA in the absence of ATP than in the presence, suggesting that, although this region might be weakly accommodating of water and ions in the closed state, it becomes much less so in the open state, further ruling out this region as a key ion conducting pathway in the open channel. Thus, the lateral portals remain the most obvious route of entry for ions based on both the available crystallographic data and results from functional studies.

The relatively large size of the lateral portals in the open state crystal structure has invited some surprise, particularly due to the degree to which these portals appear to invade the lipid bilayer, and the seeming lack of contact between intersubunit helices (Figure 2). There would appear to be little to prevent the intrusion of lipids into the ion conducting pathway, and this contention is supported in a recent study from Heymann et al. (2013). Here, molecular dynamics simulations of the open P2X4R incorporated into a lipid bilayer revealed rapid intrusion of lipid molecules into the permeation pathway, resulting in a hydrophobic barrier to ion permeation that was complete within less than 80 ns. The authors noted that a minor reorientation of the TM2 domains was sufficient to produce an open state model that retained a stable water soluble pathway through the lipid bilayer, without producing a conformation at odds with previously published functional data.

Direct functional evidence for ion entry through the lateral portals has been shown by several groups utilizing SCAM. In P2X4R, cysteines introduced at positions Glu^56^ and Asp^58^ lining the lateral portals were found to be accessible to modification by MTSET^+^, which significantly inhibited transmembrane currents in these mutants in a manner that was only reversed by exposure to the reducing agent, DTT (Samways et al., 2011). This inhibition of current was observed whether ATP was present during modification or not, implying that thiol-reactive reagents can penetrate these lateral portals even in the closed state. A cysteine substituted at position Thr^57^ of P2X1R, a lateral portal-lining residue analogous to Glu^56^ in P2X4R, was likewise found to be accessible to the large thiol-reactive molecule, N-Biotinoylaminoethyl-MTSEA, in both the closed and open states (Roberts et al., 2012a). Curiously, MTS-modification of cysteines introduced at positions Thr^57^ and Ser^59^ in P2X1R had no significant functional effect on the ATP-gated current amplitude (Allsopp et al., 2011), and the same lack of effect was reported for the analogous experiments in P2X2R (Kawate et al., 2011). Nevertheless, cysteines introduced at other lateral portal-lining positions in P2X1R and P2X2R have been shown to be accessible to and modified by thiol-reactive agents, including Gly^60^, Gly^321^, and Ile^328^ of P2X1R (Roberts and Evans, 2007; Allsopp et al., 2011), and Lys^53^, Ser^54^, Leu^327^, and Ile^328^ of P2X2R (Rassendren et al., 1997; Egan et al., 1998; Haines et al., 2001). Based on the P2X2R homology model, Ile^328^ lies between the extracellular vestibules and the lateral portals and, in agreement with the suggestion from the available crystal structural that the lateral portals enlarge substantially during gating, the mutant receptor P2X2–I328C was only modified by the large bulky thiol-reactive agent, Texas Red-MTSEA, when the channel was in the open state (Kawate et al., 2011).

The relatively large size of the lateral portals in the closed state (~12Å) suggests that they do not form an appreciable barrier to permeation (Kawate et al., 2009), a fact supported by the ability of thiol-reactive agents to access and modify cysteines introduced as deep down into the TM domain as Thr^336^ (P2X2R) even when the channel is closed (Li et al., 2010). As a result, hydrated ions and water can likely diffuse freely between the bulk solution and the extracellular vestibule regardless of whether the P2XR is gated by ATP or not. When the ATP-bound P2XR opens, the ions can then immediately enter the external mouth of the channel pore and begin their journey across the opened transmembrane pore inwards toward the cytosolic space.

## Transmembrane ion conduction pathway

By necessity, actual ion conduction across the plasma membrane proper must involve the parts of a channel protein embedded in the lipid bilayer. For most ion channels, part or all of the permeation pathway is lined by fully transmembrane spanning α-helices, with a subset of ion channels having an additional re-entrant pore loop dipping into the membrane from the extra- or intracellular side (MacKinnon, 2003). Although the initial cloning and sequence analysis of a P2XR suggested the presence of a re-entrant pore loop just extracellular to TM2 (Brake et al., 1994; Valera et al., 1995), subsequent functional studies, and now the available crystal structures, clearly demonstrate that this is not the case. The conventional wisdom has been that more than three TM helices are required in order to assemble a transmembrane pore of sufficient diameter to allow passage of ions (Spencer et al., 2002). Given that each P2XR subunit only has two putative transmembrane domains, there was a distinct possibility that both the TM1 and TM2 domains might to some extent contribute to the pore. This is in contrast to the tetra- and pentameric LGICs, the ionotropic glutamate receptors and Cys-loop channels, in which each of the four or five subunits is only required to contribute a single α-helical TM domain to the formation of permeation pathway (Keramidas et al., 2004; Traynelis et al., 2010). Over a decade prior to the successful crystallization and structural resolution of the zfP2XR, significant progress was made in determining the approximate role of these TM domains in ion conduction through this family of ion channels. Once the approximate amino acid sequences contributing to the transmembrane domains were identified (Newbolt et al., 1998; Torres et al., 1998b), SCAM was employed to identify the specific parts of these domains likely to contribute to the lining of the ion conduction pathway.

It is of particular importance to ascertain the degree of concordance between functional studies conducted on TM mutant P2XRs and the 3-dimensional crystal structures for two reasons. First, it is recognized that the lipid bilayer plays an important role in the packing and stability of membrane-spanning proteins, and so the absence of lipid in the crystal structures may have resulted in an abnormal arrangement of the TM domain helices relative to the P2XR in its native environment (see Zhou and Cross, 2013; Heymann et al., 2013). Second, there was a practical necessity to use a truncated version of the zfP2X4R.1 that lacked the cytoplasmic termini for crystallization (Kawate et al., 2009). Functional experiments suggest that the intracellular domains are of considerable importance to the normal functioning of P2XRs, and so it is important to confirm that their removal has not unduly affected the normal arrangement of the TM domains of these proteins within the lipid bilayer (Boue-Grabot et al., 2000; Ennion and Evans, 2002; Yan et al., 2008; Nicke et al., 2009; Roberts et al., 2012b; and see Costa-Junior et al., 2011). This said, as we believe will be clear from the following discussion, the available functional data actually correlates relatively well with the available crystal structure data.

### Limited contribution of TM1

**Figure 3:**
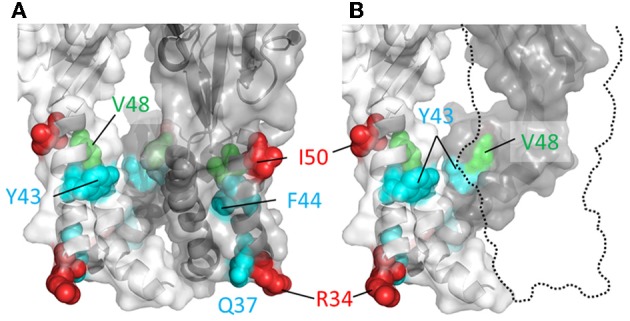
**Mapping P2X2R TM1 side-chains accessible to water-soluble thiol-reactive agents using SCAM**. TM domain regions shown with all subunits present **(A)** or the third subunit omitted **(B)**. Side chains at which cysteine substitution renders the mutant receptor sensitive to current modification by thiol-reactive agents are shown as ball models. Color coordination is as follows: Red, mutant currents modified by brief 5 s application of Ag^+^ only; Green, mutant currents modified by brief 10 s application to MTSET^+^; Cyan, mutant currents modified by prolonged (>1 min) exposure to thiol-reactive reagents.

If a part of TM1 lines the ion conduction pathway, then we would predict that cysteine substitutions introduced into at least some of the positions in this domain would be rapidly accessed and modified by water-soluble thiol-reactive agents when the channel is in the ATP-bound open state. Cysteines introduced at five positions in and near the TM1 domain of P2X2R, His^33^, Arg^34^, Ile^50^, Lys^53^, and Ser^54^, produced mutant receptors with currents sensitive to modification by short, 5 s co-applications of Ag^+^ and ATP (Haines et al., 2001) (Figure 3). Ag^+^ can coordinate thiol-groups, and its small size allows it to penetrate into small, potentially pore-lining, cavities (Lu and Miller, 1995). These side chains are located on the intra- and extracellular extremes of TM1, with Lys^53^ and Ser^54^ actually residing outside the plain of the plasma membrane, and provides little evidence that TM1 contributes significantly to the transmembrane permeation pathway proper. That cysteines introduced into TM1 are not rapidly accessible to thiol-reactive agents was later confirmed by a subsequent study which failed to observe open channel current modification in all but one mutant, P2X2R-V48C, in response to a brief 10 s exposure to the larger thiol-reactive agent, MTSET^+^ (Li et al., 2010) (Figure 3, green).

Nevertheless, although the TM1 domains do not have a primary role in forming the ion conduction pathway, they do not appear to be sealed from the water-soluble pore completely, because longer exposures to thiol-reactive agents were found to be sufficient to uncover a few additional hits within this region. Cysteines substituted into P2X2R positions Gly^30^, Gln^37^, Tyr^43^, and Phe^44^ produced mutants with currents modified by a minute long exposure to thiol-reactive agents (Jiang et al., 2001; Samways et al., 2008a) (Figure 3, cyan). The limited role of the TM1 domain in forming the narrow part of the transmembrane ion conduction path has largely been confirmed by the open channel P2XR crystal structure, which as we explain next is mostly formed by TM2 helices. That said, the extracellular extremes of TM1, including Val^48^, do appear to line the wider part of the extracellular pore opening. Indeed, from the structure it is possible to imagine how cysteines introduced at positions Tyr^43^ and Phe^44^ might have some limited accessibility to water-soluble thiol-reactive agents. These residues are located at the base the enlarged lateral portal in the open state and, although positioned far from the central axis of the conduction pathway, they may be near or possibly in contact with the water-filled cavity (Figure 3, but also see the refined P2X2R model of Heymann et al., 2013).

### Role of TM2

**Figure 4:**
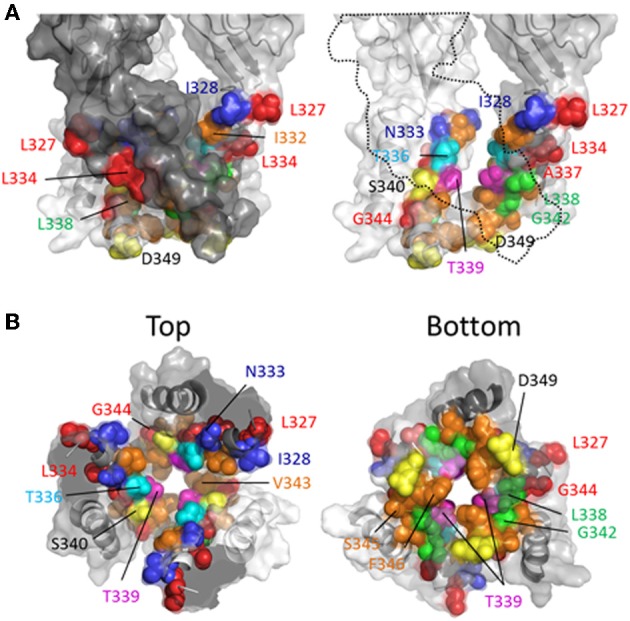
**Mapping P2X2R TM2 side-chains accessible to water-soluble thiol-reactive agents in the open state using SCAM**. Side chains at which cysteine substitution renders the mutant receptor sensitive to current modification by thiol-reactive agents applied in the open channel state are shown as ball models. **(A)** Side view of TM domains shown with all subunits present (left panel) or the third subunit omitted for clarity (right panel). **(B)** TM domains viewed from extracellular (left panel) and intracellular perspectives (right panel). Color coordination is as follows: Red, mutant currents modified by Ag^+^ only; Yellow, mutant currents modified by MTSEA^+^ only; Orange, mutant currents modified by MTSET^+^ and Ag^+^; Green, mutant currents modified by MTSEA^+^ and Ag^+^; Blue, mutant currents modified by Ag^+^, MTSEA^+^, MTSET^+^, and MTSES^−^; Cyan, mutant current modified by Ag^+^, MTSET^+^, MTSES^−^, MTS-TPAE^+^, and Texas Red-MTSEA^+^; Magenta, Ag^+^, MTSEA^+^, MTSET^+^, MTS-TPAE, but not Texas Red-MTSEA^+^ or MTSES^−^.

The TM2 domain was the initial focus of attempts to define the transmembrane ion permeation pathway for P2XRs, because early sequence analysis suggested that this region might connect with an extracellular re-entrant pore loop, not unlike that found in potassium channels (Brake et al., 1994; Valera et al., 1994). Although subsequent functional studies did not provide supporting evidence for the pore-loop hypothesis, systematic probing of the TM2 domain using SCAM presented highly compelling evidence that this was the primary pore-forming part of the P2XR subunit, which concords completely with the available crystal structures (Kawate et al., 2009; Hattori and Gouaux, 2012). In contrast to the TM1 of P2X2R, SCAM uncovered numerous hits along the length of TM2, including contiguous stretches that were confusing due to being seemingly inconsistent with this domain possessing a static helical structure (Rassendren et al., 1997; Egan et al., 1998) (Figure 4). Indeed, cysteines introduced at every single side chain between Thr^336^ and Phe^346^ have been reported to be accessible to modification by thiol-reactive compounds by one study or more (Rassendren et al., 1997; Egan et al., 1998; Li et al., 2008, 2010). However, the size of the thiol-reactive agent used and/or long durations of exposure could lead to thiol-modification of side chains that are either located in small protein cavities removed from the permeation pathway, or that are only accessible in rarely visited structural conformations.

Rassendren et al. (1997), tested the effects of three thiol-reactive agents, MTSEA^+^, MTSET^+^ and MTSES^−^ on current elicited through mutant P2X2Rs containing cysteine substitutions within TM2. Using an experimental approach in which the thiol-reactive reagents were constantly present during pulsatile ATP applications, and thus exposed to the open and closed channel conformations, it was found that >30 s long applications of MTSEA^+^ significantly modified currents through the mutant receptors, I328C, N333C, T336C, L338C, S340C, G342C, and D349C. With the exception of S340C and G342C, in which currents were potentiated by MTSEA^+^, the effect of MTSEA^+^ was inhibitory. Only three of these mutants, I328C, N333C, and T336C, also showed sensitivity to MTSET^+^ and MTSES^−^, both of which evoked inhibitory effects on channel current in each mutant (Figure 4, blue and cyan). In a later study, the acute effects of a 10 s application of MTSET^+^ on open channel currents through TM2 cysteine mutants confirmed that I328C and T336C were readily accessible to this thiol-reactive reagent (Li et al., 2008). This latter study identified several additional positions that, upon cysteine substitution, were readily accessible to MTSET^+^ in the open state within this short time frame, Ile^332^, Thr^339^, Ile^341^, Val^343^, Ser^345^, and Phe^346^. Consistent with the identification of Gly^342^, Ser^345^, and Asp^349^ in P2X2R as facing the aqueous environment, the substitution of cysteines for the analogous residues of P2X7R, Gly^345^, Thr^348^, and Asp^352^, also yielded mutants accessible to thiol-modification (Browne et al., 2013).

Investigating the stretch of TM2 between Leu^327^ and Met^356^ in P2X2R, the Egan laboratory (Egan et al., 1998) probed the accessibility of cysteines substituted into TM2 with Ag^+^, focusing on the open state of the channel in the presence of ATP. They found that ATP-gated currents through the following P2X2R mutants were rapidly modified by Ag^+^ (<5 s): L327C, I328C, N333C, L334C, T336C, A337C, L338C, T339C, G342C, V343C, G344C, S345C, L352C, and L353C. Fast Ag^+^ modification of currents mediated by N333C, T336C, A337C, T339C, V343C, and S345C were also observed in another laboratory, in addition to hits for I332C, A335C, I341C, and F346C (Li et al., 2008, 2010). It is perhaps not surprising that Ag^+^ could access and modify so many more side chains, as this relatively small ion can potentially enter narrow gaps between protein interfaces that are otherwise inaccessible to the larger thiol-reactive reagents such as MTSET^+^. We must, of course acknowledge the caveat of using SCAM that a lack of effect of a thiol-reactive agent on the current evoked through a mutant bearing an introduced cysteine is not necessarily evidence that the side chain is inaccessible to that thiol-reactive agent. Nevertheless, mapping the various hits for the different thiol-reactive agents presents a convenient pattern that seems mostly consistent with the crystal structure data (Figure 4). Thus, the three mutant P2X2Rs with currents modified by Ag^+^, MTSEA^+^, MTSET^+^, and MTSES^−^, are I328C, N333C, and T336C, which line the wide outer part of the transmembrane pore (Figure 4). The side chain of Thr^336^ is also accessible to the very large thiol-reactive agents 2-tripentylaminoethyl MTS (MTS-TPAE^+^) and Texas Red-MTSEA^+^ (Li et al., 2010). In contrast, substituting a cysteine for Thr^339^ (Figure 4, magenta) renders channel currents sensitive to Ag^+^, MTSEA^+^, MTSET^+^, and MTS-TPAE^+^, but not MTSES^−^ and Texas Red-MTSEA^+^ (Rassendren et al., 1997; Li et al., 2010), suggesting that, consistent with the open channel crystal structure, the pore narrows at this point. Cysteines substituted for Leu^338^ and Gly^342^ rendered currents sensitive only to the small thiol-reactive agents Ag^+^ and MTSEA^+^ (Figure 4, green), which fits with their off-axis orientation toward the narrow opening between intersubunit TM2 domains. Leu^334^, Ala^337^ and Gly^344^ are located in tight gaps forming the interfaces between transmembrane helices, and so it perhaps makes sense that cysteines substituted at these positions render mutant currents sensitive only to Ag^+^ (Figure 4, red).

It is interesting that thiol-modification of S340C and G342C potentiates rather than inhibits ATP-gated currents through these mutant P2X2Rs. A similar observation was made for G342C when Cd^2+^ was used as the thiol-reactive probe (Kracun et al., 2010). Both side chains are positioned slightly off the pore axis in the open crystal structure, so it is conceivable that introduction of a covalent or coordinate bonded molecule here does not obstruct the ion permeation pathway, but instead might disrupt the normal gating equilibrium of the ligand-bound receptor. In P2X7R, substitution of Gly^345^ (analogous to Gly^342^, P2X2R) produced a mutant with ATP-gated currents that were modestly inhibited by MTSEA^+^ and MTSEA-biotin applied in the open state (Browne et al., 2013), possibly indicative of subtle differences in TM domain configuration between P2XRs.

**Figure 5:**
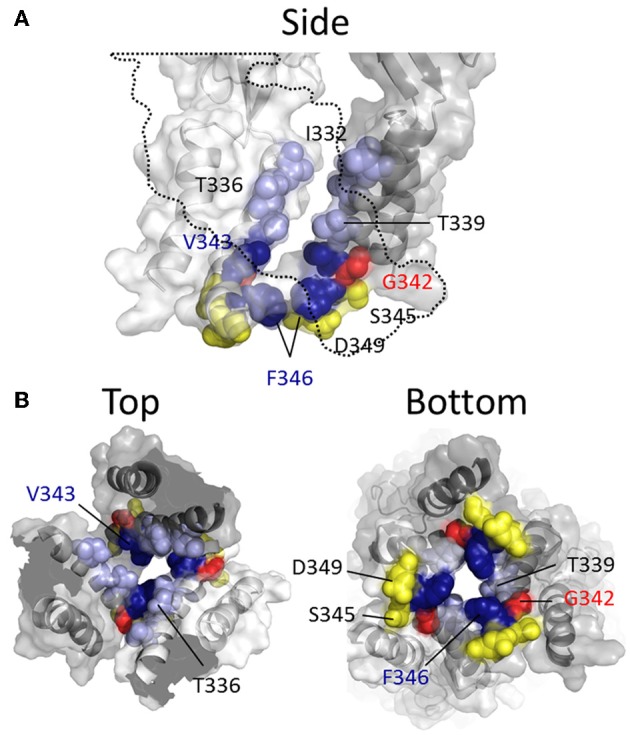
**Mapping P2X2R TM2 substituted cysteine side chains capable of coordinating Cd^2+^ in the open channel state**. Side chains at which cysteine substitution renders the mutant receptor sensitive to current modification by Cd^2+^ applied in the presence of ATP are shown as ball models. **(A)** Side view of TM domains with third subunit omitted for clarity (dotted line). **(B)** TM domains viewed from extracellular (left panel) and intracellular perspectives (right panel). Color coordination is as follows: Light blue, mutant currents reversibly inhibited by Cd^2+^; Dark blue, mutant currents irreversibly inhibited by Cd^2+^; Yellow, mutant currents irreversibly modified due to Cd^2+^ coordination between introduced Cys side chain and native amino acid side chains; Red, mutant currents reversibly potentiated by Cd^2+^.

More recent studies have employed SCAM in combination with Cd^2+^ as the thiol-reactive probe. Cd^2+^ ions can form coordinate interactions with Cys and His side chains (Kurz et al., 1995; Krovetz et al., 1997; Liu et al., 1997; Holmgren et al., 1998). Ion channel mutants with Cys substituted at positions that orientate toward the central pore axis can potentially coordinate permeating Cd^2+^, leading to current block. This can assist in identifying side chains forming the narrow regions of the transmembrane pore. Two studies identified TM2 positions at which cysteine substitutions rendered the resulting P2X2R mutant sensitive to Cd^2+^ block: I332C, T336C, T339C, G342C, V343C, D349C, and L353C (Kracun et al., 2010; Li et al., 2010), although there were some discrepancies. The mutant F346C was non-functional in Kracun et al's study (Kracun et al., 2010), whereas Li et al. successfully recorded currents from this receptor and demonstrated Cd^2+^ sensitivity (Li et al., 2010). Li et al. also reported Cd^2+^ sensitivity in the S345C mutant, which was not found to be inhibited by Cd^2+^ in the other study. Finally, Kracun et al. identified only one mutant that was irreversibly inhibited by Cd^2+^, D349C, whereas Li et al., observed irreversible Cd^2+^ block in the mutants, V343C, S345C, and F346C. Minor discrepancies aside, the combined observations of Kracun et al. and Li et al. are largely consistent with the open channel crystal structure (Figure 5). The sulfhydryl groups of neighboring Cys side chains must be ~5Å apart in order to be bridged effectively by Cd^2+^ (the coordinate bond formed between Cd^2+^ and each Cys side chain being ~2.5Å) (Dokmanic et al., 2008). Taking into account the length of the Cys side chain, ~3Å, one would predict that the backbone α-carbons of amino acid positions in which Cys substitution would favor coordination of Cd^2+^ in the central axis of the pore would need to be within ~10Å of each other. The closed P2X2R homology model shows the distance between the intersubunit α-carbons of position Val^343^ (~17Å) and Phe^346^ (~24Å) to be excessive for Cd^2+^ bridge formation. However, as the channel gates into the open conformation, these side chains come into much closer proximity, as predicted by Li et al. (2010). For Val^343^, the distance between α-carbons in the P2X2R homology models closes to ~12Å, and for Phe^346^ the distance closes to ~14Å (see Figure 7, pink). This is still not quite optimal for Cd^2+^ bridge formation, but one must consider the limitations of homology modeling, potential inaccuracies in TM arrangement as represented by the open state crystal structure (Heymann et al., 2013), and the possibility that the P2X receptor can visit open state conformations in which these side chains come into closer proximity (Kwan et al., 2012).

The α-carbons at positions Ser^345^ and Asp^349^ are positioned relatively distant from their intersubunit counterparts even in the open channel pore, at 21 and 19Å, respectively (Figure 5). However, in the case of these mutants, additional experiments conducted by Li et al. and Kracun et al., respectively, showed that Cd^2+^ coordination here might occur removed from the central pore axis, and involve additional native side chains present in the transmembrane domains. Thus, Cd^2+^ block of D349C was abolished when the native Cys^348^ adjacent to Asp^349^ was mutated to threonine (Kracun et al., 2010) (see Figure 1), whereas Cd^2+^ block of S345C was abolished by substitution of the nearby His^33^ of TM1 with tyrosine (Li et al., 2010). The effects of Cd^2+^ at these mutants, then, is not due to direct, steric blockade of ion flux due to Cd^2+^ occupying the channel pore, but may be due to an effect on channel gating.

#### Closed state specific scam hits

**Figure 6:**
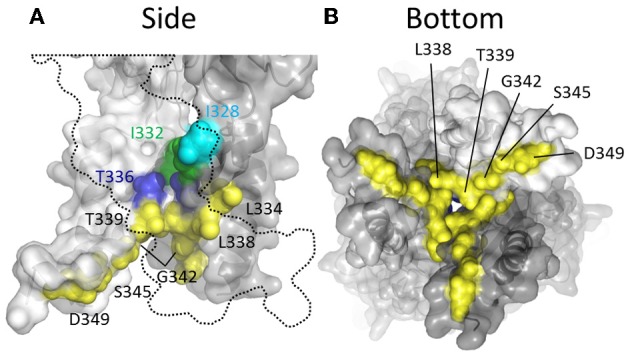
**Mapping P2X2R TM2 side-chains accessible to water-soluble thiol-reactive agents in the closed state using SCAM. (A)** Side view of TM domains. Two subunits are shown with the third omitted for clarity (dotted outline). **(B)** TM domains viewed from the intracellular side of the membrane. The colored residues indicate positions at which substitution with cysteine produces a mutant that mediates currents sensitive to modification by thiol-reactive agents applied in the closed channel state. Color coordination is as follows: Cyan, mutant currents modified by external MTSEA^+^ and MTSET^+^ applied in the closed state; Green, mutant currents modified by external Ag^+^, Cd^2+^, and MTSET^+^ applied in the closed state; Blue, mutant currents modified by external Cd^2+^ applied in the closed state; Yellow, mutant currents modified by intracellular MTSEA^+^ applied in the closed state.

Thiol-reactive agents have also been used to probe the accessibility of TM2 substituted cysteines specifically in the closed channel state of P2X2R. This involved exposure of mutants to thiol-reactive agents in the absence of ATP, and then comparing the amplitudes of subsequent ATP-gated currents to control currents evoked prior to MTS exposure. In early studies, two laboratories reported that currents through the following mutants were observed to be altered by prolonged exposure to MTSEA^+^ applied in the absence of ATP: I328C, N333C, L334C, L338C, T336C, T339C, L338C, G342C, S345C, and D349C (Rassendren et al., 1997; Egan et al., 1998) (Figure 6). However, Egan et al., observed that inclusion of free cysteine in the patch pipette solution, thereby sequestering intracellular MTSEA^+^, was found to prevent thiol-modification of currents evoked through the mutants, L334C, L338C, T339C, G342C, and S345C, suggesting that MTSEA^+^ accesses these side chains from the cytosol by first passively diffusing through the lipid bilayer in the uncharged state (Rassendren et al., 1997; Egan et al., 1998). Consistent with this, a later study also conducted in P2XR cysteine mutants revealed that the rate of current modification by Ag^+^ and MTSET^+^ was markedly reduced in the closed channel state for the mutants T336C and T339C (Li et al., 2008). In contrast, I328C and I332C were as rapidly modified by Ag^+^ and MTSET^+^ in the closed as in the open state of the channel. I332C was also found to be accessible to Cd^+^ modification in the closed channel state, as was the mutant T336C (Kracun et al., 2010) (Figure 6, green and blue). That I328C currents are modified is not surprising given their apparent location near to the lateral portals, which are wide in both the closed and open channel state. That positions Leu^338^, Thr^339^, Gly^342^, and Ser^345^ are accessible to intracellular MTS reagents is mostly consistent with the closed channel crystal structure, in which these positions can be seen to be exposed to the cytosol within the inverted cone opening out from the putative channel gate (Figure 6, yellow).

### Location of the channel gate

SCAM studies conducted prior to the publication of the closed state crystal structure for the P2XR revealed divergent hypotheses on the exact position of the channel gate in members of the ion channel family. Egan et al. (1998) observed that only intracellular MTSEA^+^ could access a Cys introduced at position Leu^334^ of P2X2R in the closed state, implying that this residue might form the intracellular extreme of the gate constriction. However, in the same study, Gly^342^ was found to be accessible by MTSEA^+^ from both sides of the membrane rendering a firm conclusion on gate position difficult. Rassendren et al. (1997) argued for a gate location between Leu^338^ and Asp^349^, finding that, in contrast to Egan et al. (1998) extracellular MTSEA^+^ could access and modify Leu^338^ in the closed channel state.

**Figure 7:**
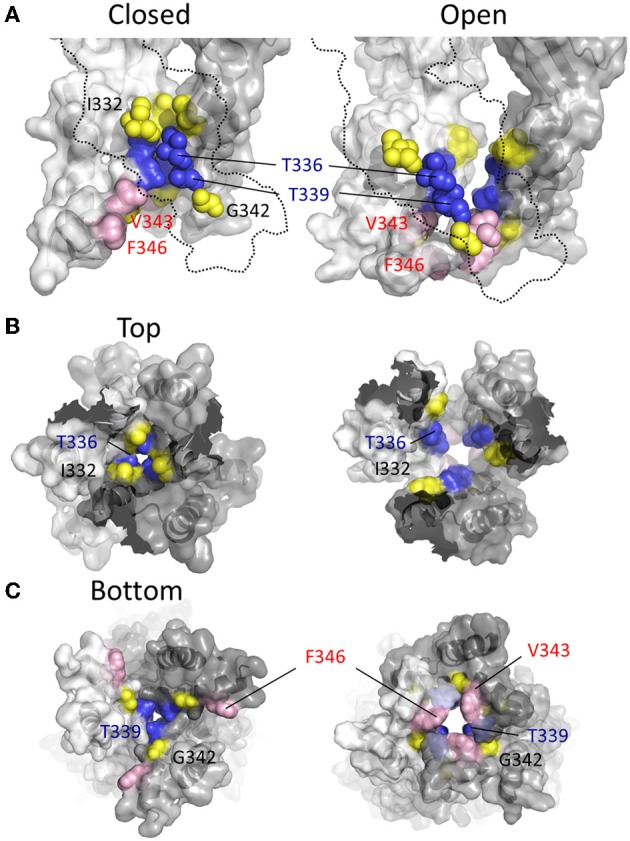
**Location of channel gate in P2X2R. (A)** Side views of TM domains in closed (left panel) and open (right panel) conformation. Residues near and within the channel gate are shown in blue and yellow for all three subunits, with the remainder of the missing subunit outlined (dotted line). The blue residues highlight the putative channel gate. Residues immediately flanking this region are shown in yellow. Val^343^ and Phe^346^, which converge upon the permeation pathway in the open state, are shown in pink. **(B)** TM domains viewed from the extracellular vestibule in the closed and open configurations. **(C)** TM domains viewed from the intracellular side in the closed and open configurations.

In two later studies, Li et al. (2008) concluded that the closed state pore narrowed to an ion impermeable constriction within the stretch of amino acids between Ile^332^ and Thr^339^ (Li et al., 2008; Kracun et al., 2010). Cysteines introduced here required progressively longer applications of MTSET^+^ for functional modification of the channel. It was observed that Ag^+^ and Cd^2+^ access to a Cys substituted for Ile^332^ was unaffected by whether the channel was opened or closed, but that Cys introduced at positions intracellular to Thr^336^ were almost completely inaccessible in the closed state, supporting the idea that the main barrier to small monovalent cations began in this region of the pore (Li et al., 2008; Kracun et al., 2010). Looking to the crystal structures, according to the closed state homology model for P2X2R the barrier to ion permeation is formed by Thr^336^ and Thr^339^ (Figure 7, blue). This agrees with the predictions of Kracun et al. (2010) and Li et al. (2008) that the extracellular side of the gate occurs just below Ile^332^ (Figure 7, yellow) and is in accordance with SCAM studies investigating the effect of Cd^2+^ and Ag^+^ on mutants P2X2Rs containing Cys substitutions within TM2 (Kracun et al., 2010; Li et al., 2010).

## Ion selection and permeation

P2XRs are commonly described as non-selective cation channels, being chiefly permeable to Na^+^, K^+^ and Ca^2+^ under physiological conditions, although at least one family member has significant permeability to Cl^−^ (North, 2002). Like other cation-permeable LGICs, at negative membrane potentials the ionic electrochemical gradients chiefly favor influx of Na^2+^ and Ca^2+^ through P2XRs, causing depolarization and an elevation in [Ca^2+^]_*i*_. The depolarization is sufficient to initiate action potential propagation (Cook et al., 1997; Dowd et al., 1998; Kirkup et al., 1999; Rong and Burnstock, 2004) and the elevation in [Ca^2+^]_*i*_ can affect a diverse array of Ca^2+^-dependent signaling processes, including synaptic transmission (Khakh and Henderson, 1998; Boehm, 1999; von Kugelgen et al., 1999; Khakh and Henderson, 2000), smooth muscle contraction (Smith and Burnstock, 2004), and cell survival (Adinolfi et al., 2005).

This review will confine itself to a very brief and simplified general introduction to the study of ion selectivity and flux through ion channels, before reviewing the current understanding of these matters with respect to P2XRs. A more thorough discussion of the general mechanisms underlying ion selectivity and flux ion channels can be obtained from other sources (Eisenman and Horn, 1983; Eisenman and Dani, 1987; Hille, 2001). The reader is also directed to reviews covering ion selectivity and flux through specific ion channels, such as the voltage-gated K^+^ (Roux, 2005), Na^+^ (Catterall, 2012), and Ca^2+^ (Sather and McCleskey, 2003) channels, and the ligand-gated Cys-loop (Sine et al., 2010), and ionotropic glutamate receptors (Traynelis et al., 2010).

The study of ion permeability and flux has a rich and prestigious history (Hille, 2001 and see Catterall et al., 2012), and the topic can be approached using a number of experimental techniques. The most common approaches involve the estimation of the “relative permeability” of ions, either from determination of current reversal potentials or from the comparison of single channel conductances in solutions of varying ionic composition. Relative permeabilities are usually reported for an ion, X, with respect to Na^+^, P_X_/P_Na_, or Cs^+^, P_X_/P_Cs_, and are calculated from experimental reversal potential and/or single channel conductance data using the Goldman-Hodgkin-Katz (GHK) voltage and current equations, respectively (Hille, 2001). Despite the fact that ion permeation through most, if not all, ion channels violates the assumptions of independence implicit to the GHK model (that permeating ions neither interact with the walls of the channel pore nor with other ions in the permeation pathway), these methods have nevertheless provided a good approximation of ion permeability for a number of cation non-selective LGICs. Nevertheless, in the case of assessing relative Ca^2+^ permeability, an alternative model-independent method is becoming more commonly used, the “fractional Ca^2+^ current” or “dye overload” method developed by Neher (1995) and Rogers and Dani (1995). This involves performing whole cell patch clamp fluorimetry with a pipette containing a saturating concentration of the Ca^2+^ dye, fura-2, and measuring both ionic current and Ca^2+^ influx simultaneously. From this, direct determination of the contribution of Ca^2+^ to the total inward current is obtained in the presence of physiological concentrations of extracellular Ca^2+^. By contrast, to determine P_Ca_/P_Cs_ using the reversal potential method commonly requires the use of non-physiological ionic solutions, and only allows permeability to be determined for a single membrane potential (the *E*_Rev_). This technique has proved particularly useful, then, for estimating Ca^2+^ flux in a physiologically normal ionic environment and at a range of membrane potentials most likely encountered by an animal cell *in vivo* (Burnashev, 1998; Jatzke et al., 2002; Egan and Khakh, 2004; Fucile, 2004; Samways et al., 2008b).

The entry of an ion into a channel pore and subsequent conductance across the biological membrane partition is influenced by a number of ion channel pore properties (see Hille, 2001). First, the maximum diameter of the channel pore will provide a steric size limit on permeating ions, allowing selection by “molecular sieving”. Second, the electrostatic environment near the openings of the pore can produce local surface potentials that contribute to selectivity by attracting and repelling ions based on charge. Lastly, the presence of charged and polar side chains, or backbone carbonyls, oriented toward the axis of narrow portions of the ion permeating pathway can directly interact with the permeating ions, assisting with dehydration, if necessary, for the ion to pass through completely.

### Upper pore size limit for P2XRs

An early rough estimate based on single channel cation conductance predicted the P2X2R pore diameter to be ~20Å at its narrowest point (Ding and Sachs, 1999a), much larger than the ~7Å predicted by the crystal structure of the open P2X4R (Hattori and Gouaux, 2012). Functional studies of cation permeability and uptake of fluorescent cationic dyes suggest that the pore diameter of P2XRs can accommodate cation species as large as the cationic dye YO-PRO1 and the large thiol-reactive agent MTS-TPAE^+^, predicting a maximum pore diameter closer to 12–14Å (Khakh et al., 1999a; Li et al., 2010; Browne et al., 2013). An explanation for the discrepancy is that the open pore has a considerable degree of flexibility, a notion supported by the phenomenon of “pore dilation” (see below), which is proposed to allow certain members of the P2XR family to increase their permeability to larger cation species (Khakh et al., 1999a; Virginio et al., 1999a). For comparison, the pore diameters of other LGICs are as follows: the nAChR, ~8Å (Albuquerque et al., 2009); NMDAR, 6–7Å (Villarroel et al., 1995); TRPV1, 10-12Å (Chung et al., 2008); and ASIC1, ~4Å (Carattino and Della Vecchia, 2012).

### Cation vs. anion selection

P2XRs are broadly described as non-selective cation channels that favor the conduction of positively charged ions, such as Na^+^, K^+^, and Ca^2+^, vs. negatively charged ions such as Cl^−^ (North, 2002). The family members P2X1R, P2X2R, P2X4R, and P2X7R all exhibit a Cl^−^ permeability relative to monovalent cations of less than 0.1 (Virginio et al., 1999b; Samways and Egan, 2007; Browne et al., 2013) (Table 1). Nevertheless, ATP-gated currents with significant Cl^−^ permeability have been recorded previously in native cells (Thomas and Hume, 1990), and some recombinant P2X5R have a P_Cl_/P_Na_ of 0.5–0.7 (Ruppelt et al., 2001; Bo et al., 2003; Samways and Egan, 2007). Exactly how the predominantly cation-selective P2XRs discriminate between cations and anions has yet to be adequately determined. Resolution of the P2X4R crystal structure revealed that the extracellular vestibule carries a net negative surface charge, primarily due to the presence of Glu^56^ and Asp^58^ (human P2X4R numbering), and free energy calculations conducted using the zfP2X4R closed structure as a model support the view that Na^+^ and Ca^2+^ entry into this domain is favored relative to Cl^−^ (Kawate et al., 2009, 2011). However, a number of observations are inconsistent with this hypothesis. Firstly, there is little correlation between the conservation of charge at positions analogous to Glu^56^ and Asp^58^ and the cation vs. anion selectivity exhibited by P2XRs. Thus, whereas the strongly cation selective channel P2X1R has Ser at both of these positions, and no neighboring acidic side chains to compensate for the loss of charge, P2X5R has an Asp at both positions but exhibits a much weaker cation vs. anion selectivity (see Figure 1). Secondly, in trying to elucidate the structural basis of Cl^−^ permeation in P2X5R, Bo et al. (2003) observed that neutralizing the positive charge at position Lys^52^, which is occupied by a neutral Gln in P2X2R and an acidic Glu or Asp in P2X1R, P2X3R, P2X4R, and P2X7R, had no effect on P_Cl_/P_Na_. Further, the complimenting mutation in P2X2R, substitution of Lys for Gln^52^, had no effect on this channel's P_Cl_/P_Na_. Although these results are curious in light of evidence that this position regulates Ca^2+^ selection and flux relative to Na^+^ in P2XRs (Samways and Egan, 2007 and see below), it seems to imply that charge selection is not a major function of the lateral portals/extracellular vestibule of the P2XR structure. A third observation that challenges the view that charge selection might happen extracellular to the transmembrane conducting pathway is that, in SCAM studies of P2X4R, negatively charged MTSES^−^ was found to access and modify substituted cysteines introduced into the extracellular vestibule at positions Glu^56^, Thr^57^, Asp^58^, and Ser^59^ of P2X4R, and as deep into the transmembrane pore as Thr^336^ and Ser^341^ of P2X2R and P2X4R, respectively (Rassendren et al., 1997; Samways et al., 2011). The modification rates were not that dissimilar to those observed for the positively charged but similarly sized MTSET^+^, suggesting that any charge selection barrier within the typical P2XR is likely to be within, or intracellular to, the channel gate. Interestingly, charge selection was observed in the central vestibule, above the extracellular vestibule, where MTSET^+^, but not MTSES^−^, was found to access and modify cysteines introduced at positions Ser^62^ and Asn^97^ of P2X4R (Samways et al., 2011). Nevertheless, the proximity of the central vestibule is clearly not preventing MTSES^−^ from intruding deep into the transmembrane pore, likely because the pore opening is too far removed from the influence of the central vestibule's surface potential.

**Table 1:** **Relative permeability and fractional Ca^2+^ current (Pf%) data for recombinant homomeric P2XRs**.

**P2XR**	**P_Cl_/P_X_+**	**P_Ca_/P_X_+**	**Ca^2+^ flux**	**References**
	**Derived from *E*_rev_**	**Derived from *E*_rev_**	**Pf%**	
P2X1R	0.09	3.6–3.9	12	Evans et al., 1996; Egan and Khakh, 2004; Samways and Egan, 2007
P2X2R	0.02	2.2–2.9	5.7	Evans et al., 1996; Virginio et al., 1998; Migita et al., 2001; Egan and Khakh, 2004; Samways and Egan, 2007
P2X3R	n.d.	1.2	3–4.8	Egan and Khakh, 2004; Samways and Egan, 2007; Ma et al., 2012
P2X4R	0.09	4.2–4.6	16	Garcia-Guzman et al., 1997; Egan and Khakh, 2004; Samways and Egan, 2007
P2X5R	0.5–0.66	n.d.	4.5	Ruppelt et al., 2001; Egan and Khakh, 2004; Samways and Egan, 2007
P2X7R	<0.1	35	4.6	Bretschneider et al., 1995; Virginio et al., 1999b; Egan and Khakh, 2004; Browne et al., 2013

*X^+^ represent either Na^+^ or Cs^+^ depending on experimental method used*.

It seems more plausible, then, that cation vs. anion selection occurs further along the ion conducting pathway, perhaps deep within the transmembrane spanning pore as has been suggested for the Cys-loop family of ion channels (Sine et al., 2010). Although this has not been investigated systematically as yet, it has been shown that substitution of Thr^339^ of P2X2R for Lys, thereby creating a symmetrical lining of positive charge deep in the open channel pore, renders the channel non-selective for Na^+^ vs. Cl^−^ (Browne et al., 2011). Further, substitution of Arg at position Thr^339^ conferred a reversal of selectivity, with this mutant displaying a modest P_Cl_/P_Na_ of ~2. However, although Thr^339^ has been previously implicated in selection between mono- and divalent cations (Migita et al., 2001; Egan and Khakh, 2004), it is very weakly conserved position among P2XR family members, being occupied by Gly in P2X1R and Ala in P2X4R for example. It is difficult, then, to draw general conclusions about its role in selection and permeation. In P2X7R, substitution of residues intracellular to the gating region also affected cation vs. anion selection, with substitution of Lys at positions Thr^348^ (Ser^345^, P2X2R) and Asp^352^ (Asp^349^, P2X2R) significantly enhancing P_Cl_/P_Na_ (Browne et al., 2013), but again it is not clear whether these positions play an important role in charge discrimination, particularly for extracellular cations. The lack of concrete data regarding the structural underpinning of cation vs. anion selectivity is a significant deficit in our understanding of P2X function and one that would be timely to address.

### Discrimination between monovalent cations

P2XRs are considered “non-selective” cation channels in large part because they do not discriminate well between small monovalent alkali ion species. Studies using the whole cell reversal potential method reported that neither P2X1R nor P2X2R could substantially discriminate between Na^+^, K^+^, Cs^+^, or Rb^+^ (Evans, 1996). These data are largely consistent with relative permeability data obtained from single channel measurements of monovalent cation conductance, where K^+^, Rb^+^, and Cs^+^ were more-or-less equally permeable, and Na^+^ only marginally less so (Ding and Sachs, 1999a). Interestingly, Li^+^ exhibited a significantly higher permeability relative to Na^+^, K^+^, Cs^+^, and Rb^+^ as assessed from whole cell reversal potentials (Migita et al., 2001), but a lower relative permeability as assessed from single channel conductance studies (Ding and Sachs, 1999a). This is consistent with the presence of an intrapore binding site with higher selectivity for Li^+^ relative to the other alkali metal ions, with the stronger interaction slowing Li^+^ conductance through the channel (Hille, 2001). There has been some difficulty in resolving the cation conductances for all the recombinant P2XRs due to the channels exhibiting very short, flickery openings. Estimates of Na^+^ conductance at membrane potentials between −100 and −150 mV are available for P2X1R [~12 pS; (Evans, 1996)], P2X2R [21–35 pS (Evans, 1996; Ding and Sachs, 1999b)], P2X4R [9–18 pS (Evans, 1996; Priel and Silberberg, 2004; Samways et al., 2011)] and P2X7R [9–13 pS (Riedel et al., 2007)]. For comparison, under similar conditions the conductances of other non-selective cation channels including the nicotinic acetylcholine receptors, glutmate-gated NMDA receptors, and capsaicin-sensitive TRPV1 receptors are 25–50 pS (Mathie et al., 1991), 20–40 pS (Stern et al., 1992), and 50–60 pS (Premkumar et al., 2002; Samways and Egan, 2011), respectively.

### Ca^2+^ selectivity and flux

All of the P2X receptor subunits confer Ca^2+^ permeability, with the selectivity of the functional channel depending on the constituent subunits (Egan and Khakh, 2004). The homomeric P2X1R and P2X4R receptors have the highest relative Ca^2+^ permeability, with reversal potential-derived P_Ca_/P_Cs_ values of ~4–5 (Evans et al., 1996; Garcia-Guzman et al., 1997; Samways and Egan, 2007) (Table 1). The use of the superior fluorimetric flux method (Neher, 1995) confirmed the ability for these receptors to conduct an appreciable Ca^2+^ influx, showing that Ca^2+^ carries 12% and 16% of the total inward currents through P2X1R and P2X4R, respectively, at −60 mV in the presence of 2 mM extracellular Ca^2+^ (Egan and Khakh, 2004; Samways and Egan, 2007) (Table 1). P2X3R has the lowest recorded relative Ca^2+^ permeability of the family, with a reversal potential-determined P_Ca_/P_Cs_ of 1.6 (Virginio et al., 1998) and a fractional Ca^2+^ current of 3–5%. This method also allowed a more accurate estimate of Ca^2+^ flux through recombinant P2X7 receptor, which are inhibited by the high external Ca^2+^ concentrations required to obtain reversal potential-based measurements of P_Ca_/P_Na_. Indeed, a previous estimate of P2X7 relative Ca^2+^ permeability from reversal potentials reported a P_Ca_/P_Na_ of 35 (Bretschneider et al., 1995), which is far in excess of what is predicted for a channel with a Pf% reading of ~5% (Egan and Khakh, 2004) (Table 1).

As expected, the variability in relative Ca^2+^ permeability exhibited between P2XR subunits correlates with a variability in the Ca^2+^ permeabilities of ATP-gated currents in native tissues. For example, in smooth muscle cells, reversal potential measurements allowed an estimation of P_Ca_/P_Na_ = 3 for P2XR-mediated currents, which is in agreement with the highly Ca^2+^ permeable P2X1R being a predominant subtype in this tissue (Benham et al., 1991). Reversal potential experiments conducted in ATP-sensitive neurons have yielded Ca^2+^ permeability values of P_Ca_/P_Cs_ = ~1.5 for nodose ganglion neurons (Virginio et al., 1998) and P_Ca_/P_Cs_ = ~2 for retinal ganglion neurons (Taschenberger et al., 1999), both cell types of which likely express homo- and heteromeric P2X2R and P2X3R. In addition, early Pf% experiments conducted in sympathetic neurons reported that, at −60 mV in the presence of 2.5 mM extracellular Ca^2+^, approximately 7% of the total ATP-gated current was carried by Ca^2+^ (Rogers and Dani, 1995). In activated, P2X4R expressing mammalian microglial cells, ATP-gated currents exhibited a Pf% value of 17% (Toulme et al., 2010), comparable to the 16% calculated for the recombinant mammalian receptor (Egan and Khakh, 2004; Samways and Egan, 2007).

In contrast to our relative lack of understanding with regard to how P2XRs discriminate between cations and anions, we have at least some idea of which amino acid side chains are important in regulating Ca^2+^ selectivity and flux through this family of ion channels. Studies using both the reversal potential and Pf% methods initially revealed an important role of polar side chains within TM2 in regulating Ca^2+^ selection in P2X2R (Migita et al., 2001; Egan and Khakh, 2004). Specifically, it was found that substitution of the polar residues Thr^336^, Thr^339^, and Ser^340^ with hydrophobic residues of similar size almost abolished the selectivity between Ca^2+^ and Na^+^ (Migita et al., 2001) and substantially reduced the Pf% (Egan and Khakh, 2004). The homology model of P2X2R is consistent with this, showing that the three residues are located in the narrow part of channel pore and that two of them, Thr^336^ and Thr^339^, are oriented directly into the permeation pathway in the open channel state (see Figures 4, 7). Indeed, increasing the side chain volume at these positions via substitution with Tyr actually formed a barrier to Ca^2+^ permeation, causing the reversal potential-based P_Ca_/P_Cs_ to be ~0.3 for the mutant T339Y, and <0.1 for S340Y (Migita et al., 2001), and the respective Pf%s recorded as ~1 and 0.2 % (Egan and Khakh, 2004) (T336Y was nonfunctional). From this, it would be tempting to speculate that the hydroxyl groups on these side chains might act as surrogate water-like ligands, assisting in the dehydration of permeating Ca^2+^ ions. For Thr^336^, this hypothesis has merit, as a hydroxyl-bearing Ser or Thr side chain resides at this relative position in all the P2X receptors with the sole exception of P2X3R (where it is occupied by Ala), P2X3R having the lowest Ca^2+^ permeability. The hydroxyl group of Thr^339^, on the other hand, is only retained in the moderately Ca^2+^ permeable P2X2R (Thr) and P2X7R (Ser), but is absent in the highly Ca^2+^ permeable P2X1R (Gly) and P2X4R (Ala). The properties of the side chain at position Ser^340^ in P2X2R are very poorly conserved, with this position most commonly being occupied by a hydrophobic residue, such as Leu (P2X4, P2X5), Ile (P2X1), Tyr (P2X7), or Trp (P2X6).

Only one negatively charged side chain is present within the actual TM domains of P2XRs, and this is a conserved Asp on the intracellular extreme of TM2 at the position analogous to Asp^349^ of P2X2R (Figure 4) and Asp^354^ (P2X4R). It is highly conserved throughout the vertebrate P2XRs, but substitution of this residue had no effect on either the reversal potential-derived P_Ca_/P_Cs_ or the Pf% of P2X2R (Migita et al., 2001; Egan and Khakh, 2004). However, Fountain et al. (Fountain et al., 2008) observed that a P2XR isoform found in green algae, which possesses an Asn at this position, exhibited a 50% increase in the reversal-potential-derived P_Ca_/P_Na_ upon substitution for Asp.

Even if a hydroxyl-bearing side chain at the position analogous to Thr^336^ of P2X2R plays a role in Ca^2+^ selectivity, it cannot easily explain the diversity in relative Ca^2+^ permeabilities between the different P2XRs. Why do P2X1R and P2X4R have such a high Ca^2+^ permeability? Inspection of the primary amino acid sequences reveals that at positions located just extracellular to TM1 and TM2, analogous to positions Glu^51^ and Asp^331^ of P2X4, some P2XRs possess fixed negative charge. Samways and Egan (2007), hypothesized that this formal charge might serve to attract divalent cations, and indeed neutralizing both charges significantly attenuated Ca^2+^ selectivity and relative conductance in P2X1R and P2X4R receptors. Complimentary mutations that substituted these charges into P2X2R, the wild type of which has Gln and Ser at these positions, significantly increased the Pf% of this receptor. Further, these acidic side chains could be titrated by reducing extracellular pH, subsequently reducing Pf% for P2X1R and for the P2X2R-Q52E/S326D mutant.

P2X3R retains a Glu at the position analogous to Glu^51^ in P2X4R and possesses an Asn at Asp^331^. Interestingly, only one of the side chains needed be present for enhanced Ca^2+^ permeability in P2X1R, P2X2R, and P2X4R receptors, and yet P2X3R receptors have the lowest Ca^2+^ permeability of the family despite retaining the Glu analogous to Glu^51^ in P2X4R. However, here the Glu is closely flanked by the positive charge of a neighboring His (as well as that of the highly conserved Lys on the other side). In addition, the lack of a hydroxyl-bearing residue at the position analogous to Thr^336^ of P2X2R might also impair Ca^2+^ permeability in P2X3R. Nevertheless, substitution of the neighboring His residue with Tyr was observed to significantly elevate Ca^2+^ permeability (Samways and Egan, 2007).

**Figure 8:**
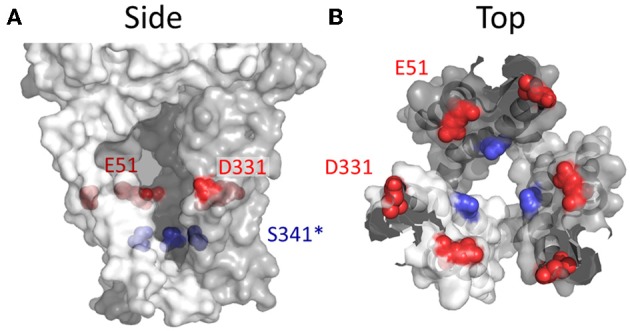
**Side chains affecting Ca^2+^ selection and flux in P2X4R. (A)** Side view of open state P2X4R homology model depicting residues known to regulate ion permeability. **(B)** P2X4R viewed from the extracellular vestibule looking intracellularly in the open configuration. Acidic side chain positions implicated in the regulation of Ca^2+^ permeation, Glu^51^ and Asp^331^ are shown in red. The position highlighted in blue is Ser^341^, a conserved hydroxyl bearing side chain predicted to regulate Ca^2+^ permeability based on data for the analogous position, Thr^336^, in P2X2R.

Glu^51^ and Asp^331^ are now known to line the lateral portals of P2X4R, and it is tempting to speculate that the presence of fixed negative charge here could present a sufficient surface potential to concentrate the activity of cations, favoring the charge dense Ca^2+^ over Na^+^ (Figure 8). However, as compelling as these data are, there are a few caveats to drawing general conclusions from them and applying them to the P2XR family as a whole. First, P2X7R has fixed charge at both of these positions, but the Ca^2+^ permeability is not quite as high as P2X1R and P2X4R (Figure 1 and Table 1). Second, P2X5R has a Lys at this position, and although this has previously been ruled out as a determinant of cation vs. anion discrimination, it seems unusual that P2X5R would maintain a Ca^2+^ permeability not far removed from that of P2X2R (Egan and Khakh, 2004) (Table 1). Third, and related to the second caveat, is the question of why lateral portal residues are sufficient to assist in discrimination between mono- and divalent cations, but not sufficient to strongly influence discrimination between anions and cations.

It might be the case that, unlike the selectivity filters of other ion channels (Sather and McCleskey, 2003; Roux, 2005; Sine et al., 2010; Traynelis et al., 2010; Catterall, 2012), P2X receptors lack a single, discreet structural locus for ion selectivity. That instead, selectivity and conductance are regulated more diffusely, and involves multiple parts of the permeation pathway. Further experiments are required to test this hypothesis. A last interesting point to make about the relative Ca^2+^ permeability of P2XRs is that it is sensitive to tuning via allosteric modulation. This was first shown for P2X1, where reducing the extracellular pH was shown to attenuate Pf%, likely as a result of charge shielding of the acidic side chains that we know confer the higher Ca^2+^ permeability in P2X1R and P2X4R (Samways and Egan, 2007). More recently, it was shown that the drug ivermectin, previously known to enhance the open probability of P2X4Rs in the presence of ATP (Khakh et al., 1999b; Priel and Silberberg, 2004), has the additional effect of reducing the Pf% of this receptor (Samways et al., 2012). Thus, far from being fixed properties of P2XRs, ion selection and conductance by these channels appears to be flexible and potentially amenable to fine tuning. In the last part of this review we will discuss a particularly unusual example of this flexibility with regard to the phenomenon described as “pore dilation”.

### Pore dilation

A curious property of P2XRs was hinted at by early studies conducted on leukocytes, where it was revealed that prolonged exposure to ATP had the effect of permeabilizing the membrane to much larger ionic species than those usually conducted by LGICs, including 2-Amino-2-hydroxymethyl-propane-1,3-diol (TRIS), N-methyl-D-glucamine (NMDG^+^) (Nuttle and Dubyak, 1994), and the cationic dye ethidium (Tatham et al., 1988; Wiley et al., 1993). In some mast cells and macrophages it was even observed that sustained ATP exposure promoted the uptake or leakage of large anionic dyes, including the Ca^2+^-sensor Fura-2 and Lucifer yellow (Steinberg et al., 1987; Yan et al., 2008). It was subsequently revealed that the purinergic receptor being acted upon by ATP in these cells was largely P2X7 (Surprenant et al., 1996). Since this discovery, sustained ATP activation of two other members of the family, P2X2R and P2X4R, has been shown to produce a similar biphasic effect on membrane permeability, causing a progressive change from a primarily Na^+^, K^+^, Ca^2+^ conducting current (I_1_) to one that conducts passage of larger cations such as NMDG^+^ and the propidium dye, YO-PRO1 (I_2_) (Khakh et al., 1999a; Virginio et al., 1999a). A fact even more curious, but one that will not be discussed further in this review, is that two members of the structurally unrelated TRP family of ion channels, TRPV1 and TRPA1, has been observed to exhibit a strikingly similar form of dynamic permeability (Chung et al., 2008; Banke et al., 2010), hinting at a broader significance of this ion channel phenomenon.

Three basic hypotheses have been put forward with regard to the mechanism by which this time-dependent change in permeability to large ionic species occurs. The first posits that the phenomenon represents an intrinsic gating property of the functional P2XR channel, and that sustained ATP exposure causes the channel pore to literally widen as the commonly used term “pore dilation” describes, thereby mediating the progressive increase in permeability to large ionic species (Khakh and Lester, 1999; Khakh et al., 1999a; Virginio et al., 1999a). The switch from I_1_ to I_2_ might be spontaneous or regulated by second messenger-mediated modification of the channel protein (e.g., phosphorylation/dephosphorylation). The second hypothesis proposes that an agonist-dependent redistribution and oligomerization of P2XRs leads to the formation of macropores. These could potentially arise from the fusion of two or more trimeric P2XRs and an enlargement of the main functional channel pore, or as a result of a separate but larger pore formed between aggregating trimeric assemblies (Khakh and Egan, 2005; Khakh et al., 1999a). The third proposes that the permeability to large cations is mediated by a structurally separate transport pathway stimulated downstream of P2XR activation (Virginio et al., 1999a). The second hypothesis has been largely ruled out by a study utilizing total internal reflection fluorescence (TIRF) imaging to monitor the lateral movement of P2X2R within the plasma membrane during ATP stimulation, which found no compelling evidence of the redistribution and clustering that would be expected if these channels were oligomerizing into a higher stoichiometric pore structure (Khakh and Egan, 2005). Which of the two remaining hypotheses holds true has been more difficult to determine, with evidence for and against both mechanisms, and the possibility remaining that the observed change in membrane permeability during sustained ATP exposure may involve both intrinsic “pore dilation” and recruitment of secondary transport pathways, and that the contribution of these two mechanisms may differ depending on cell type (for review, see North, 2002; Pelegrin, 2011).

#### Large ion permeation through the P2XR pore: I_1_ to I_2_ transition

There is a substantial body of evidence that the intrinsic pores of P2X2R, P2X4R, and P2X7R receptors can potentially accommodate large cations, including fluorescent dyes. In addition to SCAM studies showing that large MTS reagents like MTS-TPAE and even Texas Red-MTSEA can gain access to side chains deep within the permeation pathway (Li et al., 2010; Browne et al., 2011; Samways et al., 2011), site-directed mutagenesis experiments have shown that substituting side chains in the permeation pathway and intracellular domains can alter the ability of the P2XR to transition between the I_1_ and I_2_ permeability states (Khakh et al., 1999a; Khakh and Lester, 1999; Virginio et al., 1999a; Khakh and Egan, 2005; Yan et al., 2008). For P2X7R, substitutions introduced in the N-terminal domain can lock the receptor in an immediately NMDG^+^ permeable I_2_ state (Yan et al., 2008), supporting the idea that the accommodation of larger polyatomic cations is within the size limits of the intrinsic pore of these ion channels. This argument is further supported by the observation that YO-PRO1 exhibits the characteristics of a permeant blocker of Na^+^ currents when applied to the ATP-gated P2X7R (Browne et al., 2013), a result similar to one for the putatively pore dilating TRPV1R (Li et al., 2011).

Accepting that P2XRs have the capability of accommodating larger ionic species of up to 12Å in diameter (Browne et al., 2013), an important question is whether the transition from I_1_ to I_2_ during sustained activation is automatic and readily occurs in the absence of large ionic species, or whether the presence of these large species is required to induce I_2_ formation, perhaps via a “foot-in-the-door” mechanism. Two studies have published data relevant to this question, but with conflicting results. With NMDG^+^ as the chief extracellular charge carrier, Jiang et al. (2005) observed that complete pore dilation of P2X7R expressed in HEK293 cells usually occurred within 30 s of sustained ATP exposure, consistent with previous studies (Khakh et al., 1999a; Virginio et al., 1999a). However, when they exposed the cells to 30 s of ATP in the presence of normal extracellular saline, and then switched the extracellular Na^+^ for NMDG^+^ in the continued presence of ATP, they observed that the channel's permeability state started in I_1_ and then slowly shifted to I_2_. One interpretation is that NMDG^+^ needs to be present to induce the transition from I_1_ to I_2_. However, a very similar experiment was conducted in GT-1 cells expressing recombinant P2X7Rs, but in this case the substitution of NMDG^+^ for Na^+^ after 60 s of ATP exposure showed the channel to already be in I_2_ (Yan et al., 2008), suggesting that the pore widened in the absence of large cations. Establishing the mechanism by which P2XRs transition from the I_1_ to the large cation permeable I_2_ state is a worthy objective for future investigation, particularly in light of the apparent potential for using LGICs with wide pores as conduits for selective delivery of therapeutic drugs into cells (Binshtok et al., 2007; Li et al., 2011).

The hope for the future is that, in addition to the currently available closed and open state models for the P2XRs, a new model showing the putative pore dilated state will be resolved. As it is, there is already compelling evidence from crystallographic data for a similar trimeric family of ion channels, the ASIC receptors, giving credence to the idea that ion channels can occupy multiple conductance states of different diameter (Lingueglia et al., 1997; Baconguis and Gouaux, 2012). In addition, structural data suggests that the diameter of the open channel pore of the bacterial mechanosensitive channel, MscL, can change between 2 and 30Å depending on the degree to which the pore-forming transmembrane domains tilt relative to one another in the lipid bilayer (Doyle, 2004).

#### Separate downstream permeation pathways may contribute to some of the observed permeability changes

Although there is sound evidence supporting the ability for large ionic species to enter and permeate the channel of some P2XRs, this does not necessarily exclude the possibility that separate downstream permeation pathways also contribute to large ion transport. An initial piece of evidence favoring the view that the presence of other proteins, whether channel forming or not, might be necessary for the observed time-dependent changes in membrane permeability witnessed during prolonged ATP exposure was that the appearance of the phenomenon is far from consistent. Even in overexpression systems, the progressive increase in membrane permeability to large cations during prolonged ATP exposure often only occurs in a subset of the cells studied (Virginio et al., 1999a), and in other cases studies have failed to reproduce the phenomenon at all (Petrou et al., 1997; Klapperstuck et al., 2000; Pannicke et al., 2000). This may be due to the I_1_ to I_2_ transition being dependent on modification of the P2XR channel by kinases or phosphatases, but may also be due to the need for some separate pore forming protein to be co-expressed in the same cells as the activated P2XRs. In some cases, a mismatch between the observed increase in large cation permeability of P2X7R mediated currents using electrophysiology, and observed uptake of cationic dyes in intact cell imaging experiments, suggests that at least some dye uptake might occur through a non-P2X7R-mediated pathway (Virginio et al., 1997; Jiang et al., 2005). Additionally, single channel studies in macrophages revealed that prolonged ATP exposure was associated with the openings of a large conductance (~400 pS) pore permeable to large anions and cations (Faria et al., 2005), but these high conductance openings have not been observed in single channel studies of the recombinant P2X7R (Riedel et al., 2007). Furthermore, the increased permeability of macrophages to large ionic species during sustained ATP-exposure was found to be dependent on Phospholipase C-mediated elevations in [Ca^2+^]_*i*_ and MAP kinase, despite the fact that inhibition of neither of these pathways had any effect on P2X7R-mediated currents (Donnelly-Roberts et al., 2004; Faria et al., 2009).

A possible candidate for a putative secondary ion transport pathway was the gap junction-forming protein, Pannexin-1. A growing body of evidence suggests that there is a functionally significant interaction between P2X7R and Pannexin-1 (Pelegrin and Surprenant, 2006; Locovei et al., 2007; Gulbransen et al., 2012; Poornima et al., 2012; Xu et al., 2012), raising the compelling possibility that the latter is responsible for some of the observed ATP-dependent increase in membrane permeability to larger ionic species. Regardless, successive studies have shown that neither pharmacological blockade of Pannexin-1 nor inhibiting its expression affects the observed time-dependent change in permeability observed for P2X2R (Chaumont et al., 2008) P2X4R (Bernier et al., 2012), or P2X7R (Yan et al., 2008; Alberto et al., 2013). In addition, it appears that Pannexin-1 is directly inhibited by ATP within the relatively high concentration range required to activate native P2X7R receptors (Qiu and Dahl, 2009).

In cases in which prolonged ATP exposure correlates with an increase in membrane permeability to anionic species, the argument for a secondary permeation pathway independent of the P2XR pore itself is more compelling. Cankurtaran-Sayar et al. (2009) observed an ATP-mediated increase in permeability to large anions in HEK293 cells transfected with P2X7R, but found that this was due to a Ca^2+^-dependent mechanism separate from that mediating permeability to large cations. Results from a study by Schachter et al. (2008) go as far as to suggests that, in some cases, the ATP-dependent increase in permeability to anions might occur irrespective of whether there are functional P2XRs present; a result that urges caution in studying P2XR pore dilation in cell models that likely contain other purinergic receptors coupled to second messenger cascades, which might be linked to these other permeabilizing pathways. Given that Pannexin-1 channels have been shown to be permeable to large anions (Ma et al., 2012; Poornima et al., 2012), it may yet transpire that these proteins have a role to play in ATP-mediated increases in membrane permeability to large ionic species in some settings.

## Conclusion

The current understanding of ion permeation through the members of the P2X receptor family of cation permeable ion channels can be summarized as follows. Regardless of whether the P2X receptor is in the closed or open conformation, extracellular ions likely diffuse freely between the bulk solution and the extracellular vestibule of the channel by way of the three large intersubunit lateral portals. Upon ATP binding, signal transduction and channel gating, the TM domains withdraw from the central axis of the pore, iris-like, thus relieving the constriction formed by the TM2 domains in the closed channel state (Figure 6). Extracellular ions can then enter and conduct through the revealed transmembrane permeation pathway into the cell. Rather than a specific structural locus existing for ion selectivity, the data suggest that a number of sites within the open channel permeation pathway, some within the lateral portals and some deep within the narrow transmembrane pore, contribute to this essential ion channel property. Indeed, ion selection and conductance appear to be dynamic properties of P2XRs, with the ion permeable pore potentially inhabiting multiple open states with distinct permeation properties (Khakh and Lester, 1999; Virginio et al., 1999a; Samways et al., 2012).

Several questions remain, but one that is arguably of a particular pressing nature is precisely how these channels exhibit a preference for cations over anions. And extension of this question would include how some P2X receptors can become increasingly permeable to very large polyatomic cation species without apparently attenuating their ability to discriminate between small monovalent cations and anions. Another question is what role the intracellular domains play in ion permeation, whether direct by virtue of interacting with entering and exiting ions on the intracellular side of the membrane, or indirectly via effects on the arrangements of the TM domains in the open channel state.

### Conflict of interest statement

The authors declare that the research was conducted in the absence of any commercial or financial relationships that could be construed as a potential conflict of interest.

## Abbreviations

ATP: adenosine 5′-triphosphate
SCAM: scanning cysteine accessibility method
LGIC: ligand-gated ion channel.
